# Natural Killer Cell Subpopulations and Inhibitory Receptor Dynamics in Myelodysplastic Syndromes and Acute Myeloid Leukemia

**DOI:** 10.3389/fimmu.2021.665541

**Published:** 2021-04-27

**Authors:** Vlad Andrei Cianga, Lydia Campos Catafal, Petru Cianga, Mariana Pavel Tanasa, Mohamad Cherry, Phillipe Collet, Emmanuelle Tavernier, Denis Guyotat, Cristina Rusu, Carmen Mariana Aanei

**Affiliations:** ^1^ Department of Hematology, Grigore T. Popa University of Medicine and Pharmacy, Iasi, Romania; ^2^ Department of Clinical Hematology, Regional Institute of Oncology Iasi, Iasi, Romania; ^3^ Hematology Laboratory, Universitary Hospital of Saint-Etienne, Saint-Etienne, France; ^4^ Department of Immunology, Grigore T. Popa University of Medicine and Pharmacy, Iasi, Romania; ^5^ Department of Hematology, Lucien Neuwirth Cancer Institute, Saint Priest en Jarez, France; ^6^ Department of Genetics, Grigore T. Popa University of Medicine and Pharmacy, Iasi, Romania

**Keywords:** natural killer cells, inhibitory receptors, killer immunoglobulin-like receptors, natural killer cell maturation, bone marrow, myelodysplastic syndrome, acute myeloid leukemia

## Abstract

Natural killer (NK) cells are key innate immunity effectors that play a major role in malignant cell destruction. Based on expression patterns of CD16, CD56, CD57, and CD94, three distinct NK cell maturation stages have been described, which differ in terms of cytokine secretion, tissue migration, and the ability to kill target cells. Our study addressed NK cell maturation in bone marrow under three conditions: a normal developmental environment, during pre-leukemic state (myelodysplastic syndrome, MDS), and during leukemic transformation (acute myeloblastic leukemia, AML). In this study, we used a new tool to perform multicolor flow cytometry data analysis, based on principal component analysis, which allowed the unsupervised, accurate discrimination of immature, mature, and hypermature NK subpopulations. An impaired NK/T cell distribution was observed in the MDS bone marrow microenvironment compared with the normal and AML settings, and a phenotypic shift from the mature to the immature state was observed in NK cells under both the MDS and AML conditions. Furthermore, an impaired NK cell antitumor response, resulting in changes in NK cell receptor expression (CD159a, CD158a, CD158b, and CD158e1), was observed under MDS and AML conditions compared with the normal condition. The results of this study provide evidence for the failure of this arm of the immune response during the pathogenesis of myeloid malignancies. NK cell subpopulations display a heterogeneous and discordant dynamic on the spectrum between normal and pathological conditions. MDS does not appear to be a simple, intermediate stage but rather serves as a decisive step for the mounting of an efficient or ineffective immune response, leading to either the removal of the tumor cells or to malignancy.

## Introduction

Natural killer (NK) cells are particular and important components of the immune system, with a major role in the clearance of damaged, virally infected, and tumor cells (1, 2). They achieve this goal with the help of a highly diverse repertoire of germline-encoded activating and inhibitory receptors that allow NKs to recognize and target cells that lack or downregulate the expression of major histocompatibility complex (MHC) class I molecules (3). The major inhibitory receptors maintain NK cells in an unengaged state and are comprised of the killer immunoglobulin-like receptor (KIR) family and the CD94-NKG2A heterodimer, whereas the main activating receptors belong to the natural cytotoxicity receptor (NCR) family (NKp46, NKp30, and NKp44), alongside the NKG2D variant (4–6). Several NK cells functional developmental stages have been described. The CD56^bright^ CD94^hi^ CD16^−/+^ NKG2A^+^ KIR^−^ subtype is primarily involved in the secretion of cytokines and soluble amplifying factors (tumor necrosis factor α, TNFα; interferon γ, IFNγ) with pleiotropic effects, such as a high proliferative capacity, the recruitment of macrophages, the promotion of inflammation, the activation of dendritic cells, and lymphocyte priming (7, 8). The more mature CD56^dim^ CD94^med/low^ CD16^+^ NKG2A^+/−^ KIR^+^ NKs are the most numerous NK type found in peripheral blood and bone marrow and exhibit mostly cytotoxic properties, acting to clear infected, damaged, or tumor cells *via* the release of lytic granules and antibody-dependent cellular cytotoxicity (ADCC) (7–11). The expression of CD57, a marker of highly differentiated NK cells, has been correlated with a higher cytotoxic potential and long lasting memory, a feature shared with cells that participate in adaptive immunity (12–14).

Unlike CD56^dim^ NK cells, CD56^bright^ NK cells are traditionally considered to be ineffective antitumor responders that function primarily in immunomodulation (15). Previously reported data show that NK cells play a paramount role in the control of the onset and progression of hematological tumors (16).

Several mechanisms, including the downregulation of activating receptors or the upregulation of inhibitory receptors on NK cells or the modulation of their corresponding ligands on cancer cells, appear to be responsible for tumor escape from NK cell recognition in hematological malignancies (16). Myelodysplastic syndromes (MDSs) are pre-malignant, clonal, hematopoietic cell disorders that are characterized by bone marrow cell dysplasia, ineffective hematopoiesis, and multilinear cytopenias and eventually progress to acute myeloid leukemias (AML), which are life-threatening hematological malignancies with poor clinical outcomes (17–19). The pathogenesis of MDS and AML is multifactorial, including early genetic and epigenetic events and changes in the marrow microenvironment and cellular immunity. Immune evasion mechanisms allow MDS disease-initiating hematopoietic stem precursor cells (MDS-HSPCs) escape from immune system surveillance and contribute to MDS installment and the progression toward AML (20–24). NK cells play an important role in clearing leukemic cells to control disease progression and are involved in therapy response and disease prognosis (25, 26). NK dysfunction in AML arises from maturation impairment, the low expression of cytotoxic subtypes, and the downregulation of activating receptors (26–28). Data gathered from allotransplanted patients, who were mismatched for KIR-human leukocyte antigen (HLA) compared with their respective donors, showed a lower incidence of relapse due to improved graft-*versus*-leukemia response (6, 29, 30). Altered NK cell functions were observed in both MDS and AML settings, including reduced ADCC and cytolytic properties. However, the maturation profiles of NK cells during the progression of MDS and AML remain poorly studied.

The newly developed unsupervised tools for flow cytometry data analysis can be used to identify better discriminatory markers, facilitating the development of complex panels for NK cell evaluation. In addition, the subsequent processing of these data in a non-subjective manner allows for the simultaneous visualization of multiple parameters that can be applied to summarize and interpret the results (31).

The focus of this study was to evaluate the potential qualitative and quantitative modifications of the three major NK subpopulations and the expression patterns of the CD159a (NKG2A), CD158a (KIR2DL1), CD158b (KIR2DL2/DL3), and CD158e1 (KIR3DL1) inhibitory receptors under pathological conditions. We used the Principal Component Analysis (PCA), a dimensionality-reduction tool from Infinicyt software, to perform the unsupervised identification of NK cell populations (31). Unlike most studies that have focused on evaluating circulating NK cells, we have exclusively examined bone marrow aspirates, which allowed us to better understand the immunological mechanisms that might influence the NK ontogeny.

## Materials and Methods

### Patients and Controls

Bone marrow (BM) samples were collected at the Lucien Neuwirth Institute of Cancerology (Saint-Priest-en-Jarez, France) between March 2020 and July 2020 from patients newly diagnosed with MDS (n = 25) and AML (n = 8), prior to starting therapy. Normal bone marrow samples (NBM; n = 30) were obtained from patients investigated for various cytopenias such as isolated anemia [nontropical sprue (n = 2), B-12/folic acid deficiency anemia (n = 7), anemias secondary to mechanical destruction (n = 5)], isolated thrombocytopenia (drug-induced thrombocytopenia (n = 9), non-hematopoietic autoimmune-mediated thrombocytopenia (n = 7)], without morphological dysplastic changes in hematopoietic cells and without excess of blasts on cytological examination of bone marrow aspirates. Written informed consent was obtained from each patient and NBM control, as approved by the institutional procedures of the independent ethics committee and the Comité de Protection des Personnes - Ile de France (NCT03233074/17.07.2017). Patients’ characteristics are summarized in **Supplementary Table 1** and **Supplementary Figure 1**. The NBM group comprised patients aged from 10 to 90 years old (median: 64 years), while the MDS and AML cases ranged from 53 to 88 (median: 74) and from 16 to 84 years (median: 79), respectively. Thus, the NBM group considered in the study included suitable controls for both MDS and AML (**Supplementary Figure 1A**).

The 2016 World Health Organization (WHO) criteria was used to establish the diagnosis of AML and the classification of our MDS patients (**Supplementary Table 2**) (32). MDS with excess blasts (MDS-EB) type 1 (defined by 5-9% blasts in the BM) and MDS-EB type 2 (defined by 10-19% in the BM) were pooled together for statistical purposes.

### Flow Cytometry Sample Preparation

Bone marrow aspirates were collected on K2-EDTA anticoagulant, and 800,000 cells, distributed in 4 tubes, were stained with the following backbone markers: mouse anti-human fluorescein isothiocyanate (FITC)-conjugated CD57 (clone HNK-1), mouse anti-human peridinin chlorophyll protein-cyanine5.5 (PerCPCy5.5)-conjugated CD3 (clone SK7), mouse anti-human allophycocyanin (APC)-conjugated CD16 (clone 3G8), mouse anti-human phycoerythrin cyanine7 (PECy7)-conjugated CD56 (clone B159), mouse anti-human PE-conjugated CD94 (clone HP-3D9), mouse anti-human APC-H7-conjugated, CD19 (clone SJ25C1), and mouse anti-human V500-conjugated CD45 (clone HI30). Thereafter, the cells were separated into 4 tubes and further stained with one of the following markers: mouse anti-human brilliant violet 421 (BV421)-conjugated CD158a (clone HP-3E4), mouse anti-human BV421-conjugated CD158b (clone DX27), mouse anti-human BV421-conjugated CD158e1 (clone DX9), and mouse anti-human BV421-conjugated CD159a (clone 131411). The antibodies were supplied by BD Biosciences, and the optimal concentrations were set after successive titrations. Following staining, the samples were incubated for 15 minutes in the dark at room temperature (RT) and then the red blood cells were lysed with 2 ml 1× fluorescence-associated cell sorting (FACS) lysis solution (BD Biosciences, San Jose, CA, USA), followed by another 10 minutes of incubation at RT in the dark. After two successive washing steps with 2 ml phosphate-buffered saline (PBS), containing 0.2% bovine serum albumin (BSA), 0.009% sodium azide (AZ), and 0.07% ethylenediaminetetraacetic acid (EDTA) followed by centrifugation for 5 min at 400 × g, the samples were ready for acquisition on a FACSCanto II cytometer (BD Biosciences, San Jose, CA, USA) using BD FACSDiva v1.6 software. Details regarding sample preparation and the staining procedures were previously described (33).

### Multicolor Flow Cytometry (MFC) Data Analysis

Data were interpreted with the Infinicyt, v2.0 software. All samples were analyzed for the proportion of NK cells relative to the total number of lymphocytes and for the NK subsets relative to the total number of NK cells. The reliability of our data is confirmed by the fact that no statistically significant differences between cell counts (NK cells, T cells or total lymphocytes) were identified among the three analyzed groups of patients NBM, MDS and AML (**Supplementary Figures 1B–D**).

The BM aspirates have been checked for the proportions of bright CD16 neutrophils according to previously reported data (34). Samples having >24% bright CD16 neutrophils have been excluded from the study to avoid including samples with significant hemodilution.

**Figure 1:**
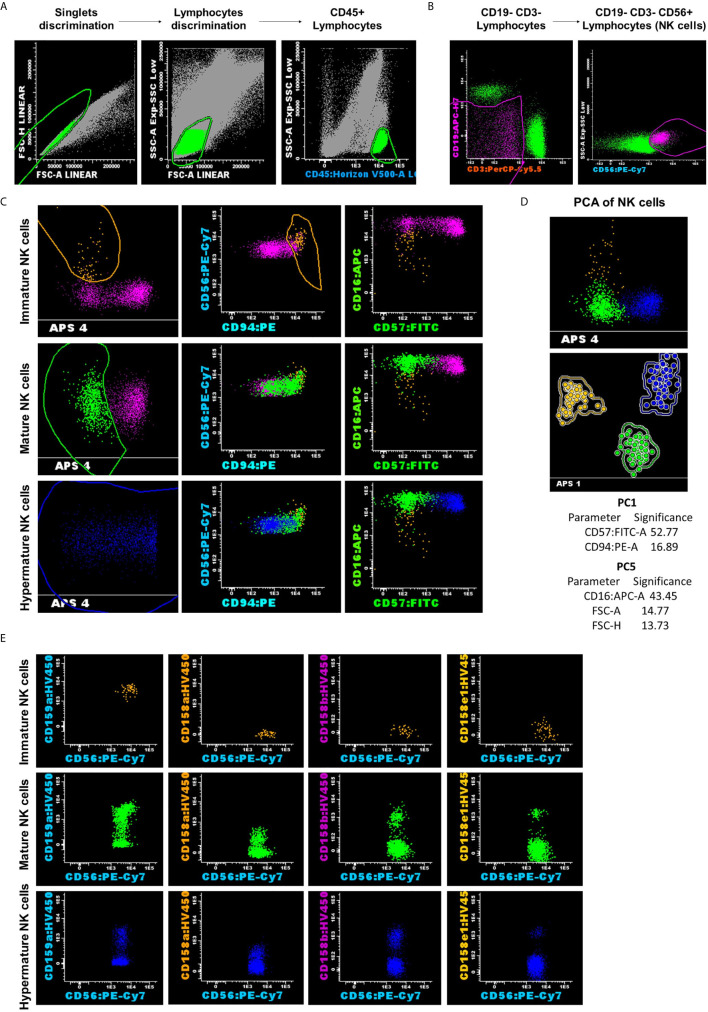
Representative example for NK analysis strategy and classification into three subsets: CD56^bright^ CD94^hi^ CD16^−^ CD57^−^ as the immature subset (yellow dots); CD56^dim^ CD94^med^ CD16^+^ CD57^−^ as the mature subset (green dots); and CD56^dim^ CD94^low^ CD16^+^ CD57^+^ as the hypermature subset (blue dots). **(A)** Lymphocytes gating on the side scatter (SSC) vs CD45 dot-plots. **(B)** NK cells were gated according to CD56 expression and the absence of CD3 and CD19. **(C, D)** Reliable separation of NK cells into subsets was obtained using the Principal Component Analysis (PCA) for all analyzed cases, regardless of the group. Dots and circles represent the median values of individual cases, and the solid line represents the 2 SD curve for an NK subset (yellow, CD56^bright^ CD94^hi^ CD16^−^ CD57^−^ represents the immature subset; green, CD56^dim^ CD94^med^ CD16^+^ CD57^−^ represents the mature subset; and blue, CD56^dim^ CD94^low^ CD16^+^ CD57^+^ represents the hypermature subset). The table shows the contribution of the most relevant parameters (those markers that received a weight over 10) to the first (PC1, x-axis) or second (PC5, y-axis) principal component reflected as percent values. **(E)** Distribution of CD159a and KIR receptors inside of the different NK cell subpopulations for the presented case.

After the exclusion of doublets and debris, lymphocytes were gated using the side scatter (SSC) *vs* CD45 dot-plots. NK cells were gated according to CD56 expression and the absence of CD3 and CD19, NK cells being defined as CD3^-^ CD19^-^ CD56^+^ events. We then selected NK subpopulations based on the expression patterns combination of CD56, CD94, CD16, and CD57 markers, using the Automatic Population Separator (APS) diagrams: CD56^bright^ CD94^hi^ CD16^−^ CD57^−^ cells were classified as the immature subgroup; CD56^dim^ CD94^med^ CD16^+^ CD57^−^ cells were classified as the mature subgroup; and CD56^dim^ CD94^low^ CD16^+^ CD57^+^ cells were classified as the hypermature subgroup (**Figure 1**). The expression patterns of the investigated NK receptors (CD158a, CD158b, CD158e1, CD159a) were evaluated for each NK cell subpopulation and for each group of cases (NBM, MDS, and AML). The fluorescence intensity of the various surface markers was evaluated using box-plots, and the number of events corresponding to each NK subgroup was exported for further statistical analysis. As the monoclonal antibodies targeting the NK receptors were conjugated with the same fluorochrome, the staining had to be performed in separate tubes. This set-up thus allowed the characterization of just one receptor at a time, and therefore it provided limited information with regard to the NK populations expressing two inhibitory receptors or more.

### Statistical Analysis

Statistical analysis was performed using Graph Pad Prism 5™ (Graph Pad Software, San Diego, CA, USA) and SPSS, v20™ (IBM SPSS Software, Chicago, IL, USA). Figures were created with Graph Pad Prism 5™. Tables, bar graphs and scatter dot plots show means with standard errors (SEM). Box-and whisker plots include the median and interquartile range without outliers. The means of normally distributed variables were statistically analyzed using the unpaired t-test and one-way ANOVA with *Post-hoc* Tukey’s Multiple Comparison test. The non-Gaussian distributed data were analyzed for median differences using non-parametric tests: Mann-Whitney test (the non-parametric counterpart to the two-sample t-test), and Kruskal-Wallis with Dunn’s Multiple Comparison test (the non-parametric counterpart to one-way ANOVA). Paired t-test and Wilcoxon signed rank test were applied for investigating the significant differences in matched parameters. One-sample t-test and Wilcoxon signed rank test (with the hypothetical value of 1.00) were used for analyzing the individual ratios of various NK cell populations. Pearson’s and Spearman’s correlation coefficients were used to assess positive associations between measured variables (age, hemoglobin value, platelet count, white blood cell count, lymphocyte count, and NK cells). The P-value below 0.05 was considered significant, and R higher than 0.5 was considered a strong correlation factor.

## Results

### Multicolor Flow Cytometry Data Gating Based on Principal Component Analysis Provides the Reliable Separation of NK Cell Subsets

The manual processing of multicolor flow cytometry (MFC) data and cell population gating using two-dimensional dot-plots can present many limitations, such as increased time-consumption, high subjectivity, and the potential to remove relevant information (35). Therefore, new algorithms for MFC data gating have been developed, including Principal Component Analysis (PCA), which draws out the underlying variance within a dataset and it is a widely used tool for visualizing multidimensional data (31). APS uses the PCA method as a compression instrument to condense relevant biological cell marker information into fewer (mostly two) dimensions (35).

The APS facilitated the classification of NK events across these three subpopulations of NK cells, particularly those belonging to the CD56^bright^ CD94^hi^ CD16^−^ CD57^−^ subset, which is the least well represented out of the three, and could easily be under- or overestimated by a biased manual analysis. Moreover, as other research groups have also stressed, the unsupervised identification of NK subsets, based on the simultaneous evaluation of the CD16/CD56/CD57/CD94 set of markers, and automatic gating is more accurate than identification methods using only one or an insufficient number of markers combined with manual interpretation (36). The strategy used in this study facilitated the reliable separation of NK subpopulations in all 3 cases of investigated groups: NBM, MDS, and AML (**Figure 1**).

### Similar Bone Marrow NK Cell Percentages in NBM, MDS and AML Conditions

The first goal was to ascertain variations in total NK cells and NK subpopulations across the different groups of cases. The mean ± standard error (SE) for the percentages of NK cells was 10.43% ± 1.34% for NBM, 13.05% ± 2.26% for MDS, and 14.23% ± 5.99% for AML samples.

Although no significant differences were observed in the NK percentages among our 3 investigated case groups (**Supplementary Table 3**, **Supplementary Figure 2A** **)**, one particular FLT3-ITD mutated AML case presented the highest proportion of NK cells out of all examined populations (55% NK cells). Within the MDS group, we identified 3 patients with >30% NK cells, whereas only one healthy control presented such a high percentage of NK cells.

### NK Cell Counts Correlate With T Cell Counts in NBM and MDS, but Not in AML

**Figure 2:**
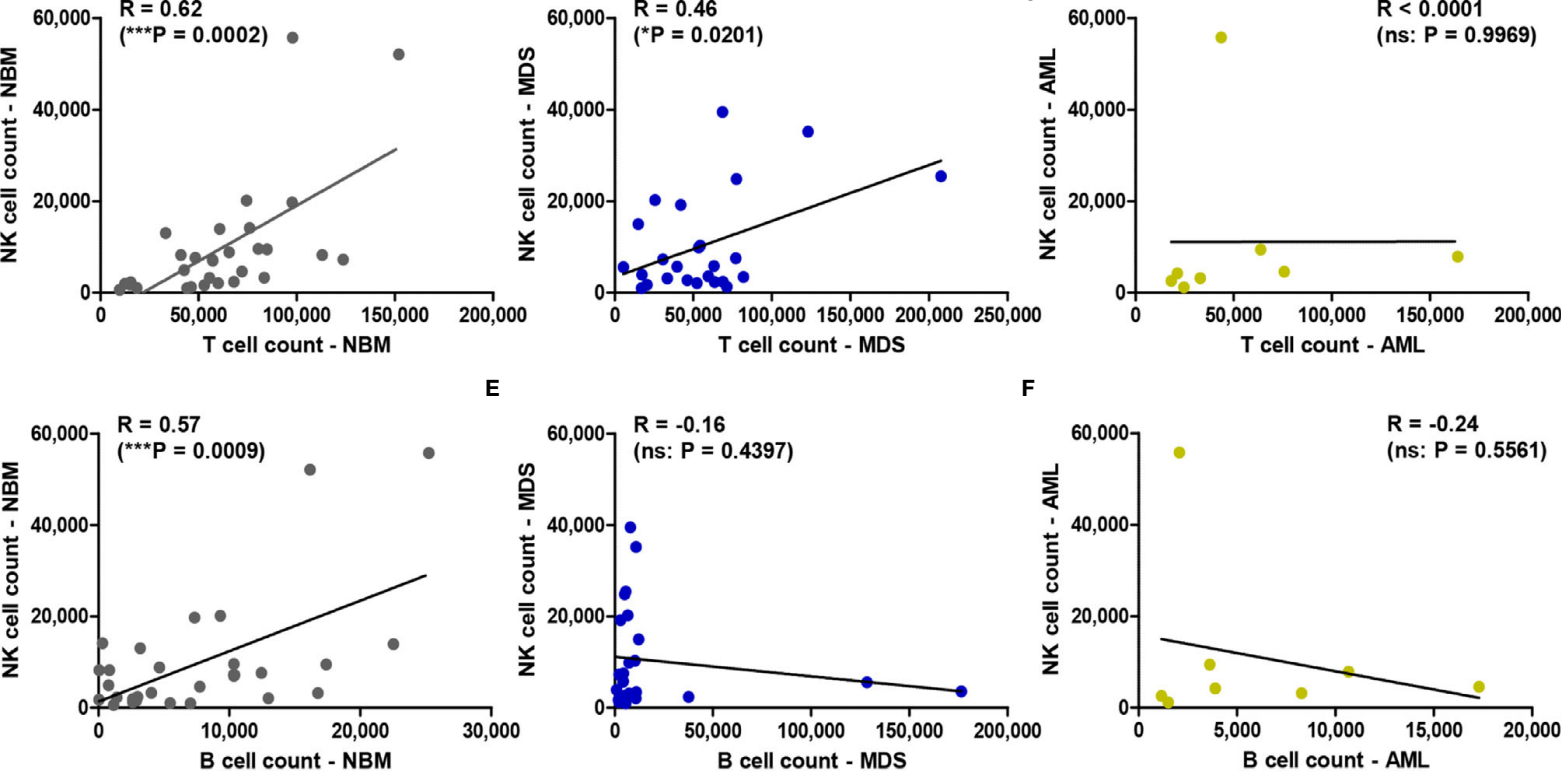
Regression statistics describing the relationship between bone marrow NK, T, and B cell counts for the three case groups: NBM, MDS, and AML. Correlation coefficients (R) were computed for **(A)** NK and T cell counts in NBM cases (n = 30); **(B)** NK and T cell counts in MDS (n = 25); **(C)** NK and T cell counts in AML (n = 8); **(D)** NK and B cell counts in NBM cases (n = 30); **(E)** NK and B cell counts in MDS (n = 25); **(F)** NK and B cell counts in AML (n = 8). Data are presented as scatter plots. Spearman test was used to analyze the significance of the identified correlations (***P < 0.001, *P < 0.05, ns, not significant).

We further explored the potential correlation between the total NK cell counts and T or B lymphocyte counts in all three investigated conditions. A strong correlation emerged between NK and T cell counts in NBM (P = 0.0002) *vs* a moderate correlation in MDS (P = 0.0201), but no correlation could be noticed in AML (P = 0.9969) (**Figures 2A–C**). As for the correlation between NK and B cell counts, a strong one was revealed in NBM (P = 0.0009) but no correlations resulted in MDS (P = 0.4397) and AML (P = 0.5561) (**Figures 2D–F**).

As regarding the cell percentages, one would expect an inverse correlation between NK cells and lymphocytes. However, an inverse correlation between NK and T lymphocytes was only seen in the NBM (R = −0.74, P < 0.0001) and AML (R = −0.92, P = 0.0009) conditions, but not in MDS (R = −0.26, P = 0.2080) (**Supplementary Figures 2D–F**). This is likely due to the increase of the B cells at the expense of NK and T cells in a number of 5 MDS cases (if compared to the highest B cell percentage identified in NBM of 23%). However, no correlation between NK and B cell percentages were observed in any of the three groups (**Supplementary Figures 2G–I**).

Further evaluations of the potential relationships between the percentages of NK cells and other parameters necessary to calculate the Revised International Prognostic Scoring System (R-IPSS) score in MDS (hemoglobin, platelets, neutrophils, and cytogenetic risk values) did not yield any strong correlations (**Supplementary Table 4**). We also evaluated whether leukemic blasts exerted a direct or indirect influence on NK differentiation in a pathologically modified bone marrow environment. Our data revealed that no correlation could be established between the percentages of bone marrow blasts and the percentages of NK cells in MDS (R = −0.37, P = 0.06) and AML samples (R = −0.28, P = 0.49) (**Supplementary Table 4**).

### Changes in Bone Marrow NK Subset Dynamics, From the Normal Setting Towards the MDS and AML Pathological Conditions

We further determined the distribution of different NK cell maturation subsets among the total NK population for each group.

**Figure 3:**
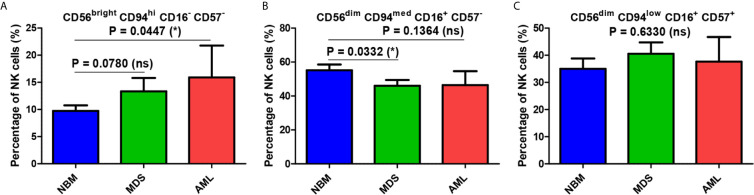
Comparison of cell percentages of distinct NK maturation subsets within the bone marrow microenvironment of NBM, MDS, and AML cases. **(A)** Percentage of bone marrow CD56^bright^ CD94^hi^ CD16^-^ CD57^-^ immature NK cells in normal bone marrow (NBM, n = 30), myelodysplastic syndromes (MDS, n = 25), and acute myeloid leukemias (AML, n = 8) cases. Bars represent the mean ± SEM (*P < 0.05, ns, not significant; one-tailed unpaired t-test). **(B)** Percentage of bone marrow CD56^dim^ CD94^med^ CD16^+^ CD57^-^ mature NK cells in NBM (n = 30), MDS (n = 24), and AML (n = 8) cases. Bars represent the mean ± SEM (*P < 0.05, ns, not significant; one-tailed unpaired t-test). **(C)** Percentage of bone marrow CD56^dim^ CD94^low^ CD16^+^ CD57^+^ hypermature NK cells in NBM (n = 30), MDS (n = 25), and AML (n = 8) cases. Bars represent the mean ± SEM (ns, not significant; one-way ANOVA followed by Tukey’s Multiple Comparison test).

A significantly increased percentage of the immature CD56^bright^ CD94^hi^ CD16^−^ CD57^−^ NK cells was observed in AML samples compared with NBM samples (P = 0.0447, **Figure 3A**). A similar increasing trend was also visible in MDS cases (P = 0.0780, **Figure 3A**). Although decreased percentages of the mature CD56^dim^ CD94^med^ CD16**^+^** CD57^−^ NK subpopulation were observed in the AML and MDS settings, only the MDS group was significantly different from the NBM control group (P = 0.0332, **Figure 3B**). No significant difference could be identified between the three groups when analyzing the hypermature CD56^dim^ CD94^low^ CD16^+^ CD57^+^ NK subpopulation (**Figure 3C**).

**Figure 4:**
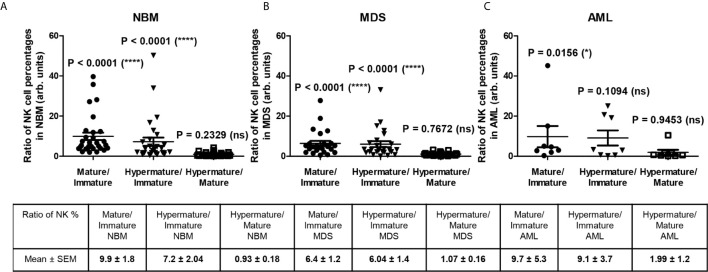
Ratio of cell percentages in distinct NK maturation subsets within the bone marrow microenvironment of NBM, MDS, and AML cases. **(A)** Ratio of cell percentages for the indicated NK maturation subsets (mature to immature, hypermature to immature, and hypermature to mature) in normal bone marrow (NBM, n = 30) cases. Data are presented as scatter dot plots, and the lines represent the mean ± SEM (****P < 0.0001, ns, not significant; Wilcoxon signed rank test). **(B)** Ratio of cell percentages for the indicated NK maturation subsets (mature to immature, hypermature to immature, and hypermature to mature) in myelodysplastic syndromes (MDS, n = 25) cases. Data are presented as scatter dot plots, and the lines represent the mean ± SEM (****P < 0.0001, ns, not significant; Wilcoxon signed rank test). **(C)** Ratio of cell percentages for the indicated NK maturation subsets (mature to immature, hypermature to immature, and hypermature to mature) in acute myeloid leukemia (AML, n = 8) cases. Data are presented as scatter dot plots, and the lines represent the mean ± SEM (*P < 0.05, ns, not significant; Wilcoxon signed rank test).

**Figure 3** reveals several significant differences that emerged when comparing the distributions of the NK subpopulations within the three groups. Furthermore, distinct patterns of maturation seem to characterize these subpopulations in the MDS and AML settings compared with those in the NBM. Statistical data showed that the NBM environment accommodated almost 7-fold more hypermature (P < 0.0001) and 10-fold more mature (P < 0.0001) than immature NK cells (**Supplementary Table 5** and **Figure 4A**). Conversely, in MDS and AML samples, the percentages of the immature NK cells gradually increased at the expense of mature NK cells (**Supplementary Table 5** and **Figures 4B, C**). Moreover, a marked heterogeneity in the ratios between hypermature and immature NK cell percentages was observed among AML cases, likely due to 3 outliers associated with the recurrent genetic mutations FLT3-ITD, IDH2, and NPM1, relative to the values for AML not otherwise specified (NOS). The AML case with the most prevalent mature NK population (69.12%) and the lowest immature NK population (1.53%) was NPM1-positive. Although NBM cases displayed a higher number of mature versus hypermature NK cells (P = 0.0086), this difference was flattened in MDS (P = 0.4502) and AML (P = 0.6066) conditions (**Supplementary Table 5**, **Supplementary Figure 3**).

### Significant Differences in NK Inhibitory Receptor Distribution Among the Immature, Mature, and Hypermature NK Cell Subsets Emerged in the MDS and AML Settings Compared With NBM Conditions

We further assessed the potential quantitative differences in the expression of NK receptors under MDS and AML pathological settings compared to NBM conditions.

**Figure 5:**
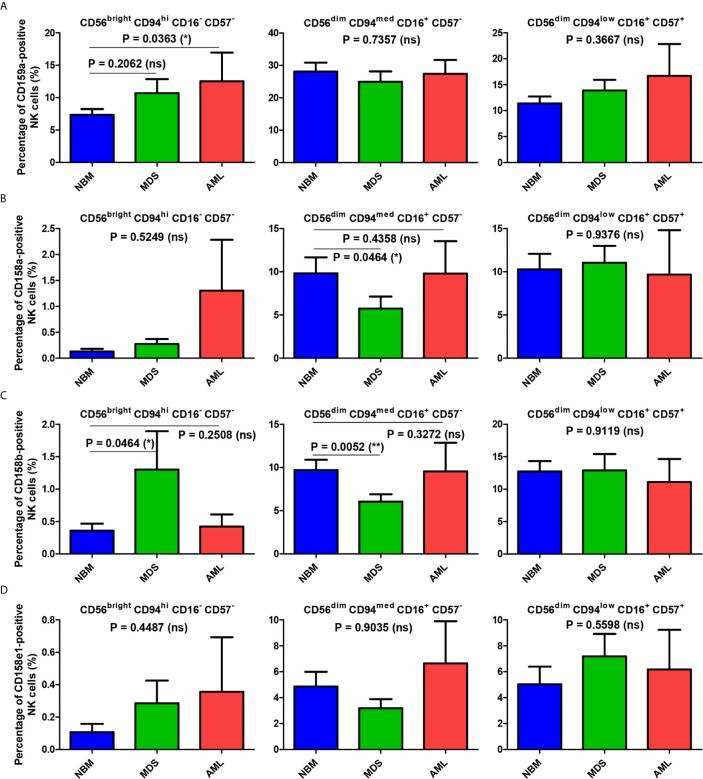
Comparison of the mean percentages of NK subsets expressing CD159a and KIR receptors within the bone marrow microenvironment of NBM, MDS, and AML cases. **(A–D)** Left panel: CD56^bright^ CD94^hi^ CD16^-^ CD57^-^ immature NK subset; middle panel: CD56^dim^ CD94^med^ CD16^+^ CD57^-^ mature NK subset; right panel: CD56^dim^ CD94^low^ CD16^+^ CD57^+^ hypermature NK subset; **(A)** Percentage of bone marrow CD159a-positive NK subsets in normal bone marrow (NBM, n = 30), myelodysplastic syndromes (MDS, n = 25), and acute myeloid leukemias (AML, n = 8) cases. Bars represent the mean ± SEM (*P < 0.05, ns, not significant; one-way ANOVA test followed by Tukey’s Multiple Comparison test; only for comparing immature NK subsets of NBM *vs.* MDS: Mann Whitney test). **(B)** Percentage of bone marrow CD158a-positive NK subsets in NBM (n = 30), MDS (n = 24), and AML (n = 8) cases. Bars represent the mean ± SEM (*P < 0.05, ns, not significant; one-tailed Mann Whitney and Kruskal-Wallis followed by Dunn’s Multiple Comparison tests for immature and mature NK subsets, one-way ANOVA followed by Tukey’s Multiple Comparison test for hypermature NK subsets). **(C)** Percentage of bone marrow CD158b-positive NK subsets in NBM (n = 30), MDS (n = 25), and AML (n = 8) cases. Bars represent the mean ± SEM (**P < 0.01, *P < 0.05, ns, not significant; Mann Whitney and Kruskal-Wallis followed by Dunn’s Multiple Comparison tests for immature and mature NK subsets, one-way ANOVA followed by Tukey’s Multiple Comparison test for hypermature NK subsets). **(D)** Percentage of bone marrow CD158e1-positive NK subsets in NBM (n = 30), MDS (n = 25), and AML (n = 8) cases. Bars represent the mean ± SEM (*P < 0.05, ns, not significant; Kruskal-Wallis followed by Dunn’s Multiple Comparison test).

In all three investigated groups (NBM, MDS, and AML), the percentage of NK cells expressing CD159a increased from the immature (CD56^bright^ CD94^hi^ CD16^-^ CD57^-^) to mature (CD56^dim^ CD94^med^ CD16^+^ CD57^-^) NK state, but then dropped in the hypermature (CD56^dim^ CD94^low^ CD16^+^ CD57^+^) NK stage only in the NBM (mature and hypermature: P <0.0001, **Supplementary Figure 4A**) and MDS (mature and hypermature: P = 0.0138, **Supplementary Figure 4B**) cases (**Supplementary Table 6** and **Figures 5A–C**). Under normal circumstances, the percentage of hypermature cells is lower than the mature one. However, when analyzing the individual AML cases, the percentage of hypermature CD159a–positive NK cells did not significantly decrease compared to the mature population (P = 0.1990, **Supplementary Figure 4C**), thus suggesting an actual increase in the number of hypermature NKG2A-expressing cells.

In contrast, the percentages of NBM NK cells expressing the individual KIRs (CD158a, CD158b or CD158e1) were considerably augmented in the mature and hypermature states as compared to the barely detectable levels in the immature population (**Supplementary Table S6** and **Figures 5B–D**).

When comparing the immature NK cell percentages measured in pathological conditions with those found in NBM, we could notice that the percentage of CD159a- or KIR-positive cells increased significantly in MDS (P = 0.0175), and in some cases of AML (**Supplementary Figure 5A**). While the maximum value for the CD159a-positive cells identified among the NBM cases was of only 16.54%, an extreme value of 40.24% was observed in one AML case characterized by NRAS deletion (**Supplementary Figure 4C**).

**Figure 6:**
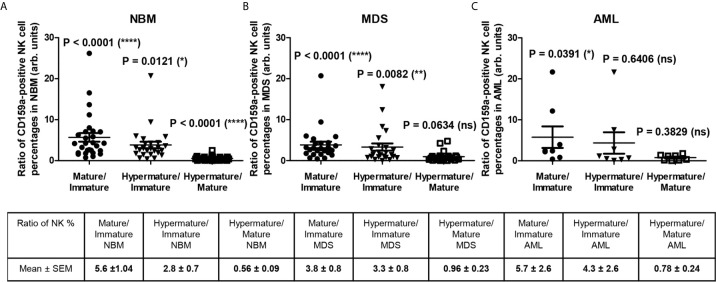
Ratio of cell percentages in distinct CD159a-positive NK maturation subsets within the bone marrow microenvironment of NBM, MDS, and AML cases. **(A)** Ratio of cell percentages for the indicated CD159a-positive NK maturation subsets (mature to immature, hypermature to immature, and hypermature to mature) in normal bone marrow (NBM, n = 30) cases. Data are presented as scatter dot plots, and the lines represent the mean ± SEM (****P < 0.0001, *P < 0.05, ns, not significant; Wilcoxon signed rank test). **(B)** Ratio of cell percentages for the indicated NK maturation subsets (mature to immature, hypermature to immature, and hypermature to mature) in myelodysplastic syndromes (MDS, n = 25) cases. Data are presented as scatter dot plots, and the lines represent the mean ± SEM (****P < 0.0001, **P < 0.01, ns, not significant; Wilcoxon signed rank test). **(C)** Ratio of cell percentages for the indicated NK maturation subsets (mature to immature, hypermature to immature, and hypermature to mature) in acute myeloid leukemia (AML, n = 8) cases. Data are presented as scatter dot plots, and the lines represent the mean ± SEM (*P < 0.05, ns, not significant; Wilcoxon signed rank test).

For the mature NK subset, we noticed a homogenous behavior among all investigated inhibitory molecules in the MDS cases, showing consistent lower percentages of NK cells expressing CD159a or KIRs (P = 0.0102), while the AML cases displayed a high heterogeneity, P = 0.8250 (**Figures 5B–D**, and **Supplementary Figure 5B**). The data regarding this significant reduction in the population of mature NK cells expressing inhibitory receptors in MDS are further supported by the individual ratio of mature to immature NK cells also showing a significant decrease compared to the corresponding normal ratio defined by the NBM cases (**Figures 6A, B**).

For the hypermature NK subset, we noticed a tendency for some of the MDS and AML cases to be characterized by extreme numbers of NK cells expressing the inhibitory molecules. While the CD159a-positive hypermature NK cells in NBM did not surpass 29%, we identified 2 MDS cases (out of n = 25) with at least 36%, and other 2 AML cases (out of n = 8) of 36% and 49% molecularly characterized by NPM1, FLT3 or FLT3-ITD mutations, respectively (**Supplementary Figure 4**). The increase of the hypermature CD159a-positive NK population in some cases of AML is also supported by the increased ratio of hypermature to immature CD159a-positive NK cells if compared to the corresponding normal ratio defined by the NBM cases (**Figures 6A, C**). For CD158a-positive hypermature NK cells, the highest cell number (43.45%) was found in one AML case characterized by recurrent mutations in NPM1 and KRAS.

In parallel, we sought to determine whether differences could be detected in terms of cellular expression intensity for these inhibitory receptors in association with different subsets of NK cells or between different groups of cases.

**Figure 7:**
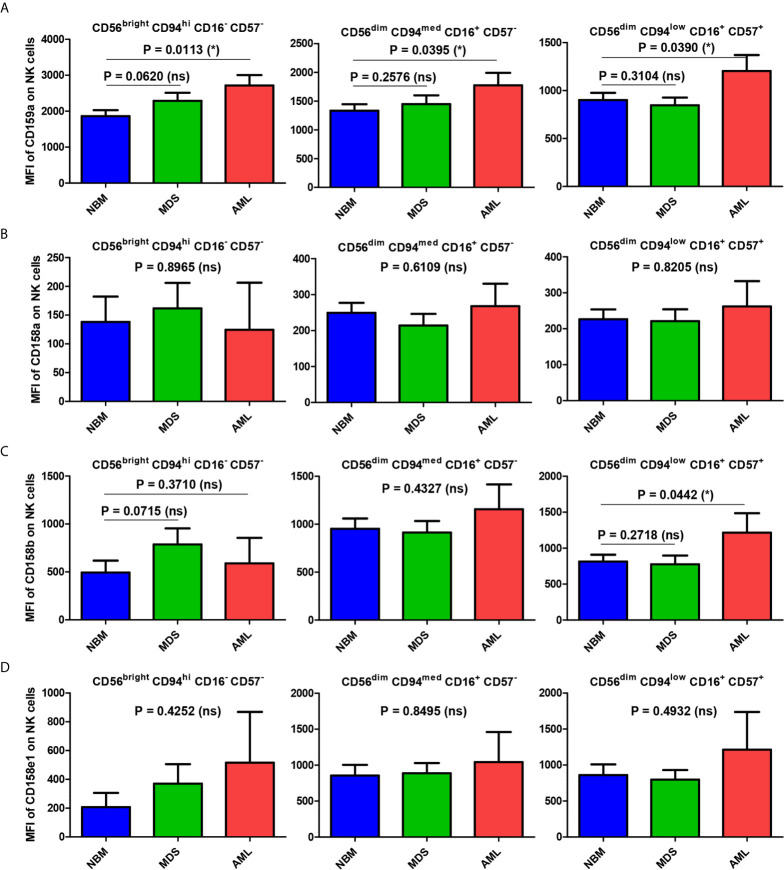
Comparison of CD159a and KIR receptors mean fluorescence intensities (MFI) of different NK maturation subsets within the bone marrow microenvironment of NBM, MDS, and AML cases. **(A–D)** Left panel: CD56^bright^ CD94^hi^ CD16^-^ CD57^-^ immature NK subset; middle panel: CD56^dim^ CD94^med^ CD16^+^ CD57^-^ mature NK subset; right panel: CD56^dim^ CD94^low^ CD16^+^ CD57^+^ hypermature NK subset; **(A)** CD159a MFI of different bone marrow NK maturation subsets in normal bone marrow (NBM, n = 30), myelodysplastic syndromes (MDS, n = 25), and acute myeloid leukemias (AML, n = 8) cases. Bars represent the mean ± SEM (*P < 0.05, ns, not significant; one-tailed unpaired t-test, and Mann Whitney test only for comparing the MFI means of mature NK subsets of NBM vs. MDS: Mann Whitney test). **(B)** CD158a MFI, **(C)** CD158b MFI, **(D)** CD158e1 MFI of different bone marrow NK maturation subsets in NBM (n = 30), MDS (n = 24), and AML (n = 8) cases. Bars represent the mean ± SEM (*P < 0.05, ns, not significant; one-way ANOVA followed by Tukey’s Multiple Comparison test).

The mean fluorescence intensity (MFI) analysis, presented in **Figure 7A** and **Supplementary Figure 6** revealed that the mean expression levels of CD159a gradually decreased from the immature (CD56^bright^) towards the mature and hypermature (CD56^dim^) NK states, in accord to literature data (14). However, the mean expression of the investigated KIRs followed a distinct pattern if compared to CD159a (NKG2A): the MFI values increased from immature to mature NK cells, and persisted at similar levels on the hypermature cells (**Figures 7B–D**). The observed MFI pattern of KIRs, but not CD159a, mirrored the cell percentages data of normal bone marrow environmental conditions.

**Figure 8:**
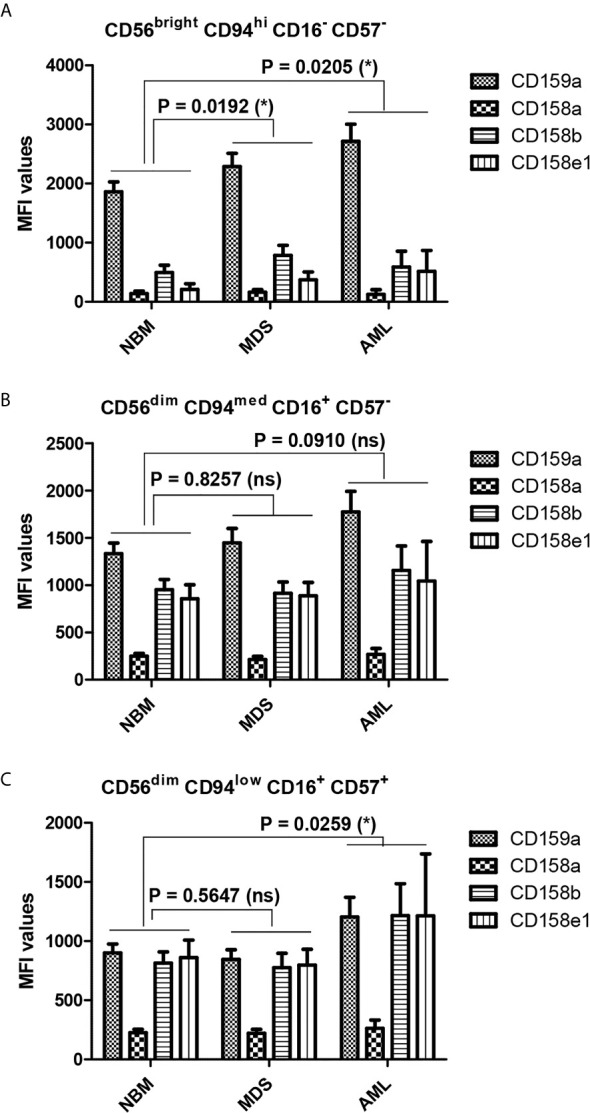
Differences in the repartition of CD159a and KIR receptors in distinct NK maturation subsets within the bone marrow microenvironment of NBM, MDS, and AML cases. **(A)** Mean fluorescence intensity (MFI) of CD159a and KIR receptors on the surface of bone marrow CD56^bright^ CD94^hi^ CD16^-^ CD57^-^ immature NK cells in normal bone marrow (NBM, n = 30), myelodysplastic syndromes (MDS, n = 25), and acute myeloid leukemia (AML, n = 8) cases. Bars represent the mean ± SEM (*P < 0.05; two-way ANOVA followed by Bonferroni post-test). **(B)** Mean fluorescence intensity (MFI) of CD159a and KIR receptors on the surface of marrow CD56^dim^ CD94^med^ CD16^+^ CD57^-^ mature NK cells in NBM (n = 30), MDS (n = 25), and AML (n = 8) cases. Bars represent the mean ± SEM (ns, not significant; two-way ANOVA followed by Bonferroni post-test). **(C)** Mean fluorescence intensity (MFI) of CD159a and KIR receptors on the surface of bone marrow CD56^dim^ CD94^low^ CD16^+^ CD57^+^ hypermature NK cells in NBM (n = 30), MDS (n = 25), and AML (n = 8) cases. Bars represent the mean ± SEM (*P < 0.05; two-way ANOVA followed by Bonferroni post-test).

When comparing the MFI data of pathological condition to NBM, we noticed a general significant increase of inhibitory molecules (except for CD158a) on MDS (P = 0.0192) and AML (P = 0.0205) immature NK cells (**Figure 8A**). The higher expression phenotype was further maintained in the mature and hypermature NK populations in AML, but not MDS (**Figures 8B, C**). However, among all inhibitory molecules, only CD159a consistently followed this general inhibitory behavior in AML (**Figure 7A**).

## Discussion

NK cells are effectors of utmost importance for the protection of an organism against malignantly transformed cells. They derive from common lymphoid progenitor cells and are now considered to belong to the innate lymphoid cells (ILCs), a group of cells that lack antigen receptors but parallel the major known T cell subsets. However, unlike T cells, ILC differentiation commences in the bone marrow environment, and many information accumulated in the recent years regarding the required transcriptional factors (many of which are also characteristic to the T cell lineage), specification and commitment stages, and cytokine input (3, 37, 38).

Surprisingly, most studies have examined peripheral blood NK cells, whereas very few have explored the bone marrow when considering AML. In one such study, Chretien et al. investigated the cryopreserved peripheral blood mononuclear cells and concluded that a correlation exists between NK cell maturation and the clinical outcomes of AML patients (27).

Our study targeted bone marrow NK cells in both normal settings and pathological conditions; therefore, the impacts of leukemic blasts and an altered microenvironment on the differentiation and maturation of NK cells should be accounted for in this study (27, 36, 39). Leukemic blasts display antigen-presenting cell-like features, modulating T cell differentiation and further impacting the activation and differentiation of many other immune cells (40–42).

Unlike previously reported data (43), we were unable to identify any correlations between the percentages of bone marrow blasts and NK cells in MDS and AML samples. This might be explained by the relatively small number of MDS and AML patients included in our study, and by the clinically and biologically heterogeneity of MDS cases as reflected by WHO criteria and the Revised International Prognostic Scoring System.

A strong correlation between the NK and T cell counts emerged in NBM and MDS, but not in the AML samples from our study. At first glance, this might be perceived as a more efficient response on behalf of the immune system in MDS than AML, meant to augment cytotoxic effectors. However, this association should be interpreted with caution, as an increase in the numbers of bone marrow T cells might not indicate an increase in T cytotoxic or T helper cells but could be associated with an increase in regulatory T cells (Tregs), as shown by others (40–42).

As the NK cell phenotype began to be characterized, CD16 and CD56 were found to be useful for distinguishing between various stages of maturation, which also translated into distinctive effector behaviors associated with changes in cytokine secretion, cytotoxic effects, and the ability to migrate (44, 45). New markers emerged, allowing for the more sophisticated characterization of various NK subpopulations. Based on the currently accepted panel of NK markers, which included CD16, CD56, CD57, and CD94, we were able to identify and quantify immature, mature, and hypermature NK cell subsets.

In recent years, the unsupervised, automatic discrimination of NK subsets has largely been used to reduce the strong heterogeneity observed in NK cell maturation profiles among patients. In our study, the APS, a PCA based analysis, facilitated the fast and reliable identification of three distinct profiles: the immature CD56^bright^ CD94^hi^ CD16^−^ CD57^−^ population, the mature CD56^dim^ CD94^med^ CD16^+^ CD57^−^ population, and the hypermature CD56^dim^ CD94^low^ CD16^+^ CD57^+^population. Furthermore, similar to the results generated by the recently published FLOCK algorithm, developed for multidimensional cytometry data analysis (36), we observed a rather heterogeneous expression of the investigated inhibitory receptors. This should be perhaps interpreted in the wider context of NK cells expressing other inhibitory molecules as well (46), which were not analyzed in this study, such as LIR/ILT2, KIR2DL4, KIR2DL5, KIR3DL2, IRp60, and p75/AIRM1 (Siglec7).

Our data illustrated no significant differences in the total percentages of NK cells between our three groups; only the in-depth analysis of AML cases revealed a heterogeneous distribution, with extreme values, as represented by the cases with FLT3-ITD, NPM1, and IDH2 mutations. However, should NK cells be investigated as a unique population, this data might offer different and erroneous conclusions.

Our study further aimed to quantify and compare the NK subpopulations in fresh bone marrow samples derived from normal individuals, AML patients, and patients with the MDS pre-malignant condition, which has been considered to represent an intermediate stage towards AML. Our data showed that although the percentage of immature NK cells in the bone marrow was significantly increased in the AML group compared with the NBM group, no such evolution could be observed for mature and hypermature NK cells. These rather unexpected results might support the conclusions reached by Chretien et al., in which the malignant bone marrow microenvironment, heavily influenced by the presence of leukemic blasts, determines whether a maturation blockade occurs (27). Mamessier et al. suggested that the de-differentiation of the NK cells (47) may occur, which is reflected in the patients’ outcomes. Hence, we should stress that this approach, which addressed the NK maturation subsets, offers a much more accurate image of the interactions between the immune system and malignant cells.

We also examined the expression patterns of several inhibitory receptors. Because MDS is viewed as an intermediate stage, a consistent pattern of evolution, starting with NBM, going through MDS, and culminating with AML, was expected. However, our data demonstrated that no such pattern emerged, and the inhibitory receptors (NKG2A and KIRs) appeared to follow distinctive evolutionary pathways depending on the NK subgroup and their presence in a normal or pathological setting.

For instance, in the NBM population, CD159a is expressed by a maximum number of cells belonging to the mature stage, but the percentage falls dramatically during the hypermature stage. In contrast, although the percentage of immature NK cells expressing KIR is extremely low (less than 1% of total NK cells), it increases markedly in the mature and hypermature populations, with no significant differences between them (10% of total NK expressing CD158a/b and 5% being positive for CD158e1). CD159a-positive NK cells linearly increase among the immature population from NBM towards AML, while no statistically significant differences between the numbers of mature and hypermature effector NK cells expressing CD159a were observed between NBM and pathological conditions. However, AML cases showed increased heterogeneity of CD159a expression in the hypermature NK subset, with the highest values being attributed to cases with the FLT3-ITD and, respectively IDH2 mutations. The significant increase in the number of CD159a-positive cells observed among immature NK cells in AML samples if compared to NBM samples, suggests a rather early response which may drive the augmentation of the numbers of NK cells displaying inhibitory receptors. This hypothesis is further strengthened by the observed increase in the numbers of hypermature CD159a cells (increased ratio of hypermature to immature NK cells) in AML samples compared with NBM samples. Interestingly, the percentage of KIR-positive immature NK cells increases in both MDS and AML compared to NBM. A somewhat homogenous pattern can be distinguished for the mature subset as the percentages of NK cells expressing the investigated receptors significantly decrease in the MDS population and increase again in the AML population, generally surpassing the values recorded for the NBM population. In hypermature NKs, similar to CD159a, the percentages of KIR-positive NK cells tend to slightly increase in MDS and are more heterogenous in AML.

When analyzing the MFI data, the level of CD159a expression decreased gradually, as expected (14) from the immature (CD56^bright^) towards the hypermature (CD56^dim^) NK subset in all three groups of cases: NBM, MDS, and AML. It thus describes a distinct pattern if compared to NK cell percentages, as the percentages of NK cells expressing CD159a increase from immature to mature and fall back towards the hypermature state. However, for the immature NK subset, the CD159a MFI data follow a similar pattern with the cell percentages observations: significantly higher expression in AML than NBM. The higher CD159a expression is further maintained in the mature and hypermature subsets in AML. Instead, for KIR receptors, the MFI data correlate with cell percentages in NBM: surface expression increases from immature to mature NK cells, with no further change toward the hypermature stage. We might thus conclude that, as the percentage of CD159a positive NK cells tends to decrease in the normal bone marrow during the transition from the immature to the hypermature stage, the inhibitory effects seems to be compensated by an increase of distinct inhibitory KIRs.

This observation suggests distinct inhibitory pathways required for each NK maturation step: while the CD159a provides the strongest inhibitory effect in immature NK cells, the KIRs dominate the inhibitory pathway in the hypermature state, thus leaving the mature cells under the influence of both pathways. For MDS cases, CD159a has a higher expression only on the immature NK cells, but it decreases to normal levels as the NK cells mature. In contrast, most of AML cases only slowly lose the inhibitory effect of CD159a during the NK cells progress from the immature towards the hypermature state. As a result, the MFI of the mature NK subset is similar to the NBM immature NKs, and the MFI of hypermature NK subset is similar to the NBM mature NKs.

Interestingly, the two AML cases associated with the NPM1 genetic anomaly (deletion or recurrent mutations) presented a remarkable increase in the fraction of mature and hypermature NK cells in the detriment of immature cells, accompanied by the highest expressions of CD159a- and CD158a-positive hypermature NK cells. The two cases of AML associated with FLT3 genetic anomalies (deletion or mutation) phenocopied part of the NPM1-associated phenotype, presenting increased number of hypermature NK cells (if compared to the immature subset) with high expression of CD159a.

These data might suggest that variations in the number of NK cells expressing a particular KIR in normal bone marrow microenvironment conditions is accompanied by corresponding variations of receptor expression levels in the respective NK subpopulations. Additionally, the above-described pattern of immature NK cell percentages was similar to the MFI data of KIR receptors: increased expression in both MDS and AML compared to NBM. For the mature and hypermature NK subsets, a clearly higher expression of KIR (similar to CD159a) was observed in AML compared to NBM, providing further evidence for an immune response that is hindered from acting against malignant cells. Interestingly, for the MDS cases, the cellular expression of CD159a and KIRs on mature NK cells was similar to NBM, despite a significant reduction in the respective cell percentages. Thus, we might conclude that MDS does not appear to represent a smooth transitional stage towards malignancy, but rather a decisive step for the evolution of the immune response to cancer cells as it struggles to choose an efficient path.

## Standard Biosecurity and Institutional Safety Procedures

All the standard biosecurity and institutional safety procedures are adhered. The laboratory has biosafety level 1 (BSL-1) standard where all standards and protocols are adopted as per the guidelines of CLSI.

## Data Availability Statement

The original contributions presented in the study are included in the article/**Supplementary Material**. Further inquiries can be directed to the corresponding author.

## Ethics Statement

The studies involving human participants were reviewed and approved by Comité de Protection des Personnes - Ile de France (NCT03233074/17.07.2017). The patients/participants provided their written informed consent to participate in this study.

## Author Contributions

Study design, VC, CA, PCi, LC. Sample preparation, VC. Data acquisition, VC. Data analysis, VC, CA, MC. Statistical analysis, MT. Clinical investigation, PCo, ET, DG. Manuscript drafting, VC, CA, PCi, MT. Manuscript revision, VC, CA, PCi, LC, MT, CR, MC. All authors contributed to the article and approved the submitted version.

## Funding

This research received funding from the “Les Amis de Rémi” Foundation. VC was supported by an Erasmus PhD mobility exchange program funded by the University of Medicine and Pharmacy “Grigore T. Popa” Iasi, Romania (131/4.11.2019).

## Conflict of Interest

The authors declare that the research was conducted in the absence of any commercial or financial relationships that could be construed as a potential conflict of interest.
