# Supplementary material for: Natural Killer Cell Subpopulations and Inhibitory Receptor Dynamics in Myelodysplastic Syndromes and Acute Myeloid Leukemia
Source: Front Immunol. 2021 Apr 27;12:665541. doi: 10.3389/fimmu.2021.665541 (PMC8112610; doi:10.3389/fimmu.2021.665541)
Supplement: Supplementary file 1 [file DataSheet_1.pdf]

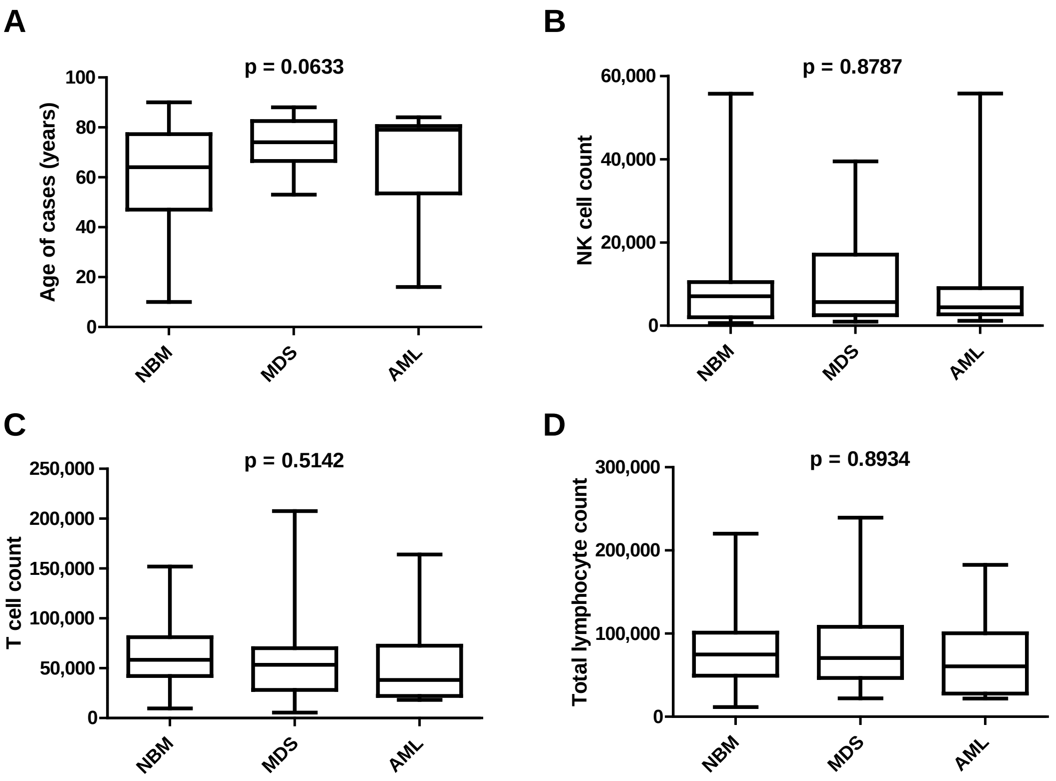

Supplementary Figure 1. Comparison of age and bone marrow cell counts for the three groups of cases analysed in this study: NBM, MDS, and AML.

(A) Age data presented as box and whisker plots for normal bone marrow (NBM), myelodysplastic syndromes (MDS), and acute myeloid leukemias (AML) cases.

(B) NK cell count data presented as box and whisker plots for normal bone marrow (NBM), myelodysplastic syndromes (MDS), and acute myeloid leukemias (AML) cases.

(C) T cell count data presented as box and whisker plots for normal bone marrow (NBM), myelodysplastic syndromes (MDS), and acute myeloid leukemias (AML) cases.

(D) Total lymphocyte count data presented as box and whisker plots for normal bone marrow (NBM), myelodysplastic syndromes (MDS), and acute myeloid leukemias (AML) cases.

Statistical calculations of significance were performed using the Kruskal-Wallis and Dunn's Multiple Comparison test with significance set for $P < 0.05$.

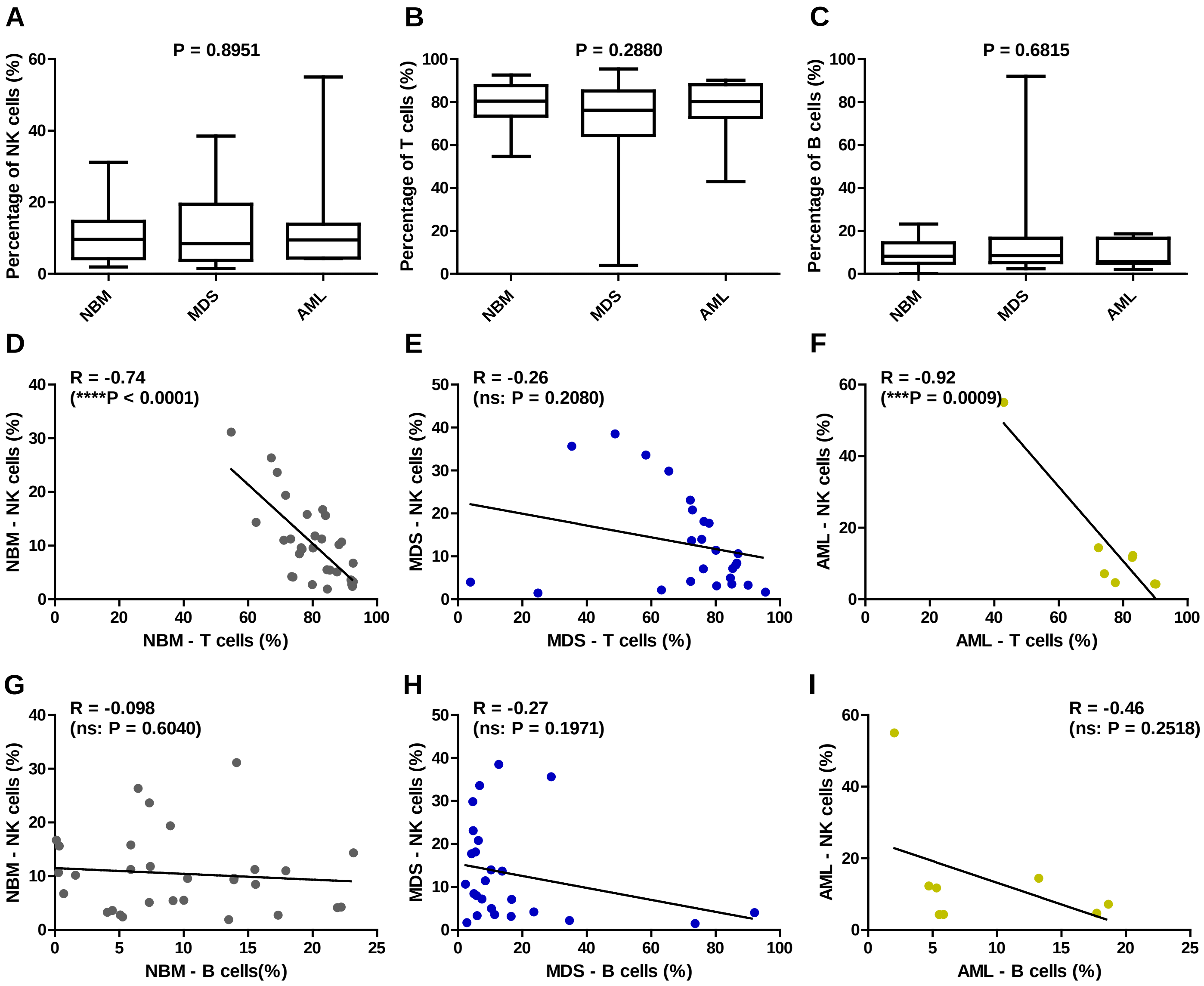

Supplementary Figure 2. Correlations between NK, T, and B cell percentages from the bone marrow microenvironment of NBM, MDS and AML cases.

(A) NK cell percentage data presented as box and whisker plots for normal bone marrow (NBM), myelodysplastic syndromes (MDS), and acute myeloid leukemias (AML) cases.

(B) T cell percentage data presented as box and whisker plots for normal bone marrow (NBM), myelodysplastic syndromes (MDS), and acute myeloid leukemias (AML) cases.

(C) B cell percentage data presented as box and whisker plots for normal bone marrow (NBM), myelodysplastic syndromes (MDS), and acute myeloid leukemias (AML) cases.

(A-C) Statistical calculations of significance were performed using the Kruskal-Wallis and Dunn's Multiple Comparison test with significance set for P < 0.05.

(D-I) Correlation coefficients (R) were computed for **(D)** NK and T cell percentages in NBM cases (n = 30); **(E)** NK and T cell percentages in MDS (n = 25); **(F)** NK and T cell percentages in AML (n = 8); **(G)** NK and B cell percentages in NBM cases (n = 30); **(H)** NK and B cell percentages in MDS (n = 25); **(I)** NK and B cell percentages in AML (n = 8). Data are presented as scatter plots. Spearman test was used to analyse the significance of the identified correlations (****P < 0.0001, ***P < 0.001, ns – not significant)

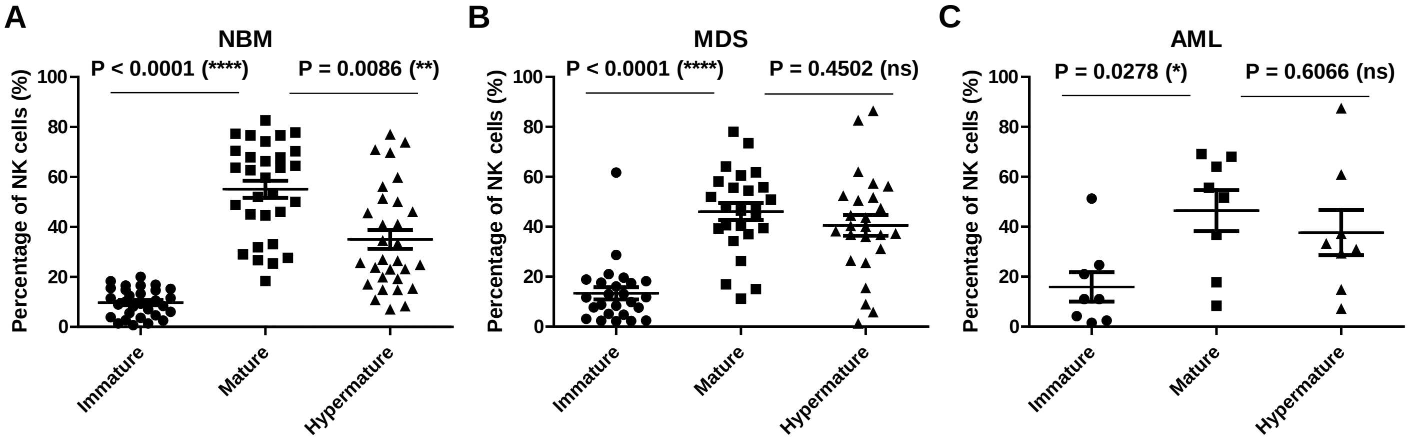

Supplementary Figure 3. Distribution of NK maturation subsets within the bone marrow microenvironment of NBM, MDS, and AML cases.

Percentages of bone marrow $CD56^{bright}CD94^{hi}CD16^{-}CD57^{-}$ immature, $CD56^{dim}CD94^{med}CD16^{+}CD57^{-}$ mature , and $CD56^{dim}CD94^{low}CD16^{+}CD57^{+}$ hypermature NK cells in **(A)** normal bone marrow (NBM, n = 30), **(B)** myelodysplastic syndromes (MDS, n = 25), and **(C)** acute myeloid leukemias (AML, n = 8) cases. Data are presented as scatter dot plots, and the lines represent the mean ± SEM (****P < 0.0001, **P < 0.01, *P < 0.05, ns – not significant; two-tailed paired t-test was applied for all cases with the exception of comparing the means of immature and mature MDS cases, when the Wilcoxon signed rank test was used).

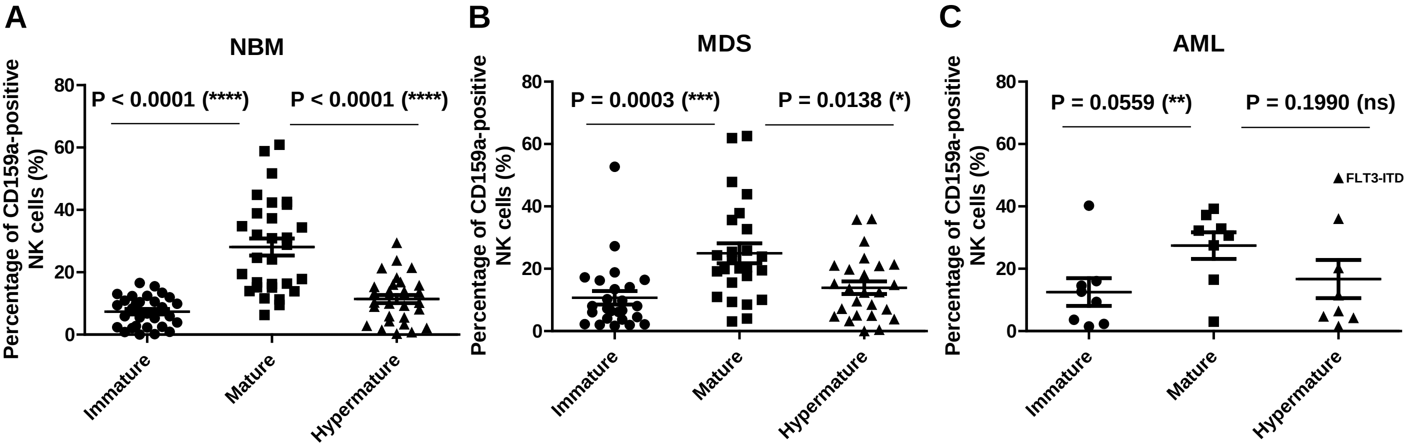

Supplementary Figure 4. Distribution of CD159a-positive NK maturation subsets within the bone marrow microenvironment of NBM, MDS, and AML cases.
Percentages of bone marrow $CD56^{bright}CD94^{hi}CD16^{-}CD57^{-}$ immature, $CD56^{dim}CD94^{med}CD16^{+}CD57^{-}$ mature , and $CD56^{dim}CD94^{low}CD16^{+}CD57^{+}$ hypermature CD159a-positive NK cells in **(A)** normal bone marrow (NBM, n = 30), **(B)** myelodysplastic syndromes (MDS, n = 25), and **(C)** acute myeloid leukemias (AML, n = 8) cases. Data are presented as scatter dot plots, and the lines represent the mean ± SEM (****P < 0.0001, ***P < 0.001, **P < 0.01, *P < 0.05, ns – not significant; two-tailed paired t-test was applied for all cases with the exception of comparing the means of immature and mature MDS cases, when the Wilcoxon signed rank test was used).

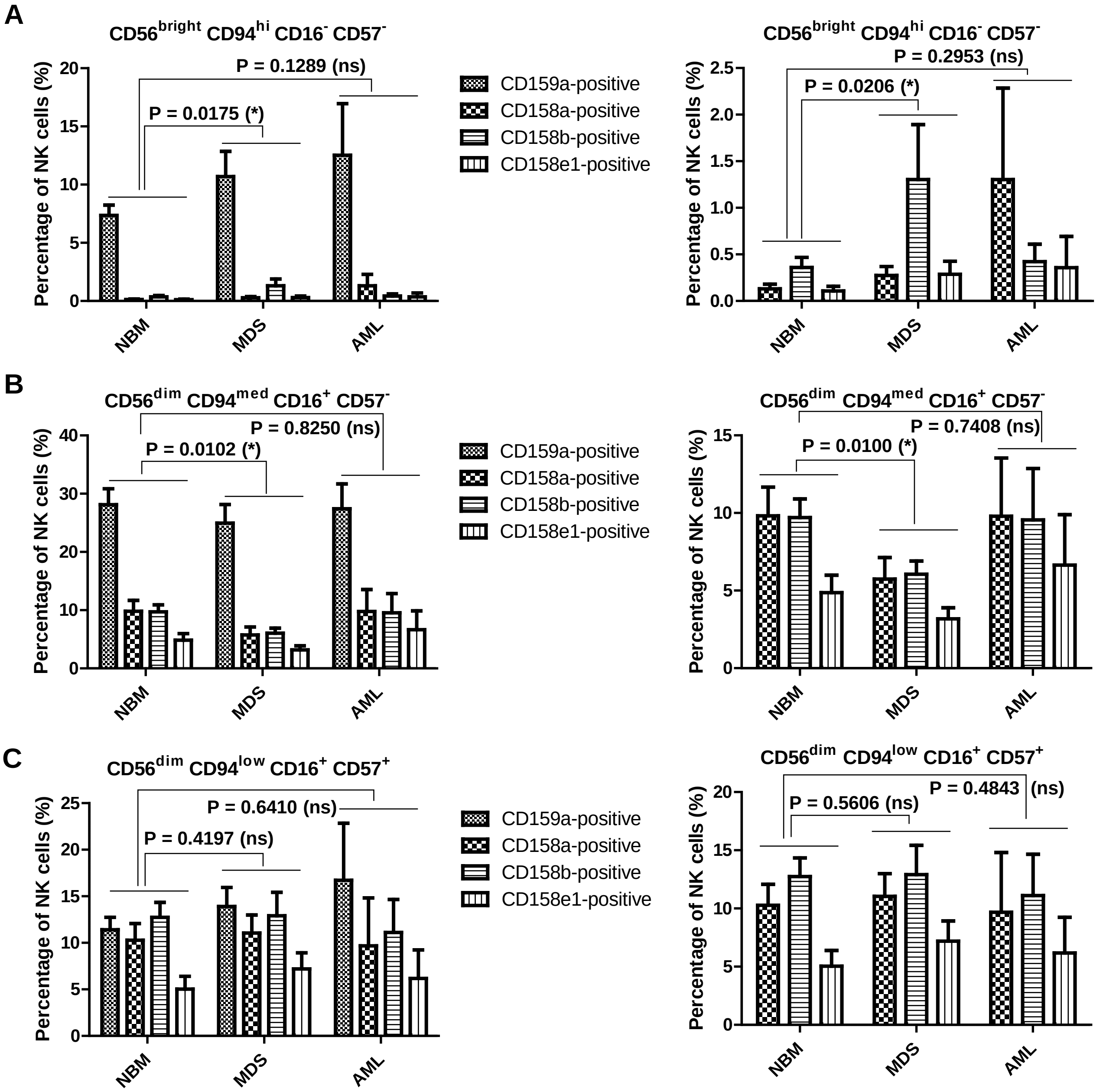

Supplementary Figure 5. Differences in the repartition of NK maturation subsets within the bone marrow microenvironment of NBM, MDS, and AML cases.

Percentage of bone marrow **(A)** CD56^{bright}CD94^{hi}CD16^{-}CD57^{-} immature NK cells, **(B)** CD56^{dim}CD94^{med}CD16^{+}CD57^{-} mature NK cells, **(C)** CD56^{dim}CD94^{low}CD16^{+}CD57^{+} hypermature NK cells expressing CD159a (left panel only) and KIR receptors (both panels) in NBM (n = 30), MDS (n = 25), and AML (n = 8) cases. Bars represent the mean ± SEM (*P < 0.05; two-way ANOVA on ranks followed by Bonferroni post-test).

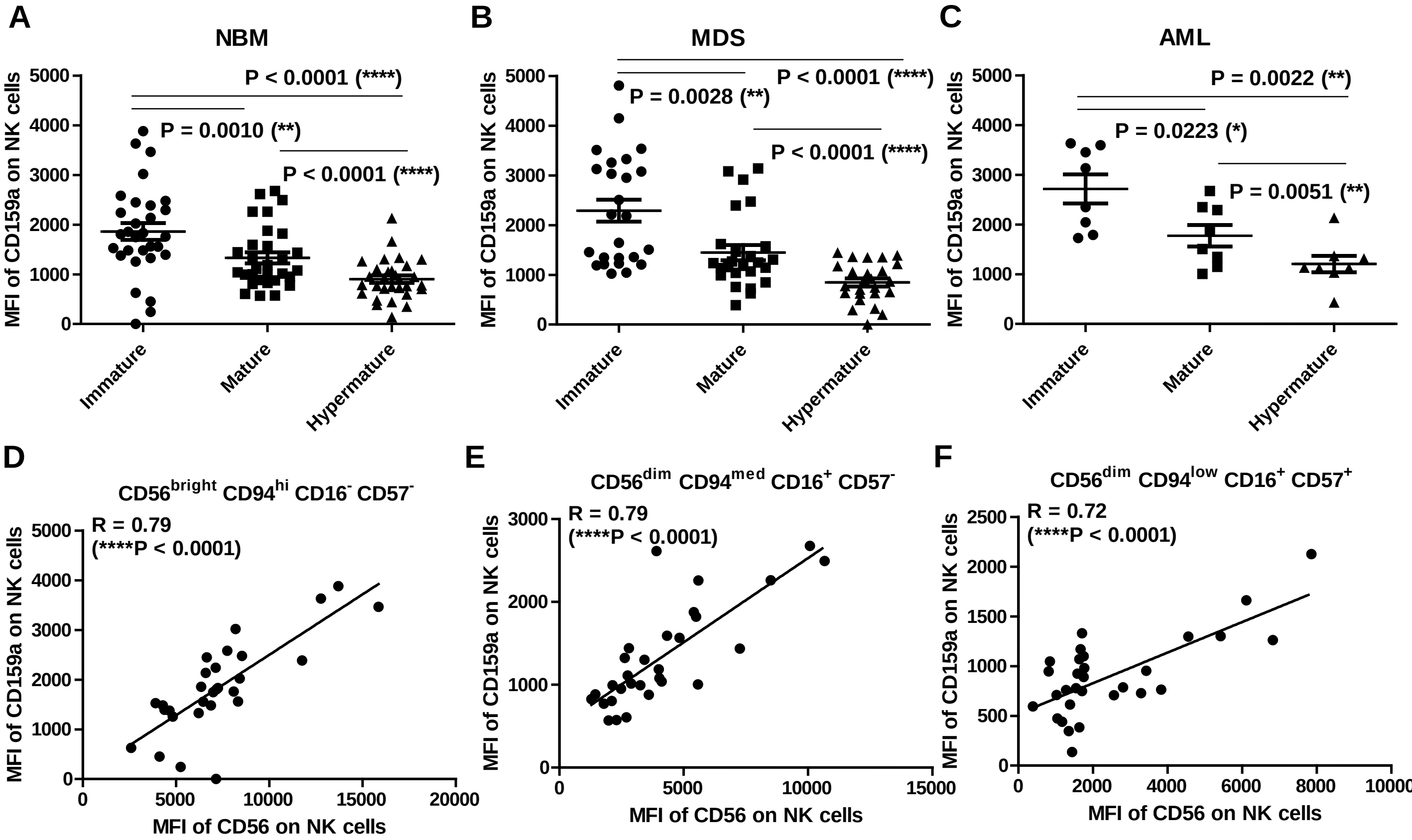

Supplementary Figure 6. CD159a MFI of distinct NK maturation subsets within the bone marrow microenvironment of NBM, MDS, and AML cases.

(A-C) Mean fluorescence intensity (MFI) of CD159a on bone marrow $CD56^{bright}CD94^{hi}CD16^{-}CD57^{-}$ immature, $CD56^{dim}CD94^{med}CD16^{+}CD57^{-}$ mature , and $CD56^{dim}CD94^{low}CD16^{+}CD57^{+}$ hypermature CD159a-positive NK cells in **(A)** normal bone marrow (NBM, n = 30), **(B)** myelodysplastic syndromes (MDS, n = 25), and **(C)** acute myeloid leukemias (AML, n = 8) cases. Data are presented as scatter dot plots, and the lines represent the mean ± SEM (****P < 0.0001, **P < 0.01, *P < 0.05, ns – not significant; two-tailed paired t-test).

(D-F) Correlation of CD159a and CD56 MFI values (R) in NBM (n = 30) for each bone marrow NK maturation subset: **(D)** $CD56^{bright}CD94^{hi}CD16^{-}CD57^{-}$ immature, **(E)** $CD56^{dim}CD94^{med}CD16^{+}CD57^{-}$ mature , and **(F)** $CD56^{dim}CD94^{low}CD16^{+}CD57^{+}$ hypermature (****P < 0.0001, F-test).

Table S1. Patients' characteristics

Characteristic	Control group	MDS	AML
**Patients no.**	30	25	8
**Age, years Mean ± SD**	60 ± 22.6	73.7 ± 9.5	67.2 ± 23.7
**Male**<br>**Female**<br>**Ratio Male/Female**	15 (50%)<br>15 (50%)<br>1	17 (68%)<br>8 (32%)<br>2.1	5 (62.5%)<br>3 (37.5%)<br>1.6
**Hemoglobin Mean ± SE**	11.22 ± 0.44 g/dl	9.89 ± 0.38 g/dl	9.825 ± 0.57 g/dl
**Platelets Mean ± SE**	167 ± 15.17 $x10^9/L$	175.2 ± 24.3 $x10^9/L$	91.75 ± 31.49 $x10^9/L$
**Leucocytes Mean ± SE**	4.260 ± 0.33 $x10^9/L$	6.488 ± 1.07 $x10^9/L$	13.51 ± 4.9 $x10^9/L$
**Lymphocytes Mean ± SE**	1.270 ± 0.13 $x10^9/L$	1.983 ± 0.69 $x10^9/L$	2.509 ± 0.91 $x10^9/L$
**T Lymphocytes Mean ± SE**	79.86 ± 1.73 $x10^9/L$	70.27 ± 4.32 $x10^9/L$	76.62 ± 5.34 $x10^9/L$
**B lymphocytes Mean ± SE**	9.65 ± 1.23 $x10^9/L$	16.6 ± 4.33 $x10^9/L$	9.13 ± 2.27 $x10^9/L$
**% of blasts on bone marrow Mean± SE**	1.97 ± 0.26	4.050 ± 0.71	52.88 ± 9.29

SD, standard deviation; SE, standard error; MDS, Myelodysplastic Syndromes; AML, Acute Myeloid Leukemia

Table S2. The Myelodysplastic Syndromes patient's stratification was assigned according to the 2016 WHO Criteria and the Revised International Prognostic Scoring System

**WHO subtypes (n, %)**	MDS-SLD 10 (40%)	MDS-RS 3 (12%)	MDS-isolated 5q deletion 0	MDS-MD 6 (24%)	MDS-EB 5 (20%)	MDS - Unclassifiable 1 (4%)
**IPSS-R risk group (n, %)**	Very low 7 (28%)	Low 9 (36%)	Intermediate 5 (20%)	High 3 (12%)	Very High 1 (4%)	
**IPSS-R prognosis survival (n, %)**	8.8 years 4 (16%)	5.3 years 9 (36%)	3 years 5 (20%)	1.6 years 6 (24%)	0.8 years 1 (4%)	
**IPSS-R AML evolution (n,%)**	Not reached 4 (16%)	10.8 years 11 (48%)	3.2 years 5 (20%)	1.4 years 2 (8%)	0.7 years 1 (4%)	

MDS, Myelodysplastic Syndrome; WHO, World Health Organization, **MDS-SLD**, MDS with single lineage dysplasia; **MDS-RS**, MDS with ringed sideroblasts; **MDS-MD**, MDS with multilineage dysplasia; **MDS-EB**, MDS with excess blasts; **IPSS-R**, Revised International Prognostic Scoring System.

Table S3. Distribution of NK cells maturation subsets in NBM, MDS and AML groups

ME (mean) ± SE (standard error)	NBM	MDS	AML
**% $CD56^{hi}CD94^{hi}CD16\text{-}CD57\text{-}$**	9.7 ± 1.0	13.34 ± 2.45	15.9 ± 5.86
**% $CD56^{dim}CD94^{med}CD16\text{+}CD57\text{-}$**	55.13 ± 3.41	46.05 ± 3.36	46.42 ± 8.2
**% $CD56^{dim}CD94^{low}CD16\text{+}CD57\text{+}$**	35.04 ± 3.78	40.58 ± 4.13	37.65 ± 9.05

Normal bone marrow (NBM), myelodysplastic syndromes (MDS), acute myeloid leukemia (AML)

Table S4. **Pearson correlation coefficients (R) between the percentage of NK cells and the parameters required for computing the R-IPSS score in MDS.**

		Age	Hemoglobin	Platelets	Neutrophils	Bone marrow blasts (%)	Cytogenetic risk
NBM	NK (%)	R = 0.1<br>P = 0.59	R = -0.02<br>P = 0.87	R = -0.01<br>P = 0.94	R = -0.05<br>P = 0.79	N/A	N/A
MDS	NK (%)	R = -0.15<br>P = 0.46	R = 0.09<br>P = 0.63	R = -0.08<br>P = 0.69	R = -0.24<br>P = 0.23	R = 0.37<br>P = 0.06	R = 0.21<br>P = 0.29
AML	NK (%)	R = 0.01<br>P = 0.49	R = 0.52<br>P = 0.18	R = 0.13<br>P = 0.74	R = -0.12<br>P = 0.76	R = -0.17<br>P = 0.66	N/A

No statistically significant correlations were identified by ANOVA F test for the investigated parameters.

Table S5. Distribution of NK cells maturation subsets in NBM, MDS and AML groups

ME (mean) ± SE (standard error)	NBM	MDS	AML
**% CD56^{bright}CD94^{hi}CD16-CD57-**	9.7 ± 1.0%	13.34 ± 2.45%	15.9 ± 5.86%
**% CD56^{dim}CD94^{med}CD16+CD57-**	55.13 ± 3.41%	46.05 ± 3.36%	46.42 ± 8.2%
**% CD56^{dim}CD94$^{med/low}$CD16+CD57+**	35.04 ± 3.78%	40.58 ± 4.13%	37.65 ± 9.05%

An increase in NK percentages was observed as maturation shifted from the immature to the mature compartment, with a subsequent decrease as the hypermature state is reached, although still in higher percentages than in the immature subpopulation.

Table S6. Percentage of positive NK cells for NKG2A (CD159a) and KIR inhibitory receptors detected in bone marrow microenvironment from NBM, MDS and AML settings

	CD159a	CD158a	CD158b	CD158e1
$CD56^{bright}CD94^{hi}$CD16-CD57- (Percentages)				
NBM	7.4% ± 0.9%	0.1% ± 0.1%	0.4% ± 0.1%	0.2% ± 0.1%
MDS	10.7% ± 2.2%	0.3% ± 0.1%	1.3% ± 0.6%	0.1% ± 0.1%
AML	12.5% ± 4.4%	1.3% ± 0.1%	0.4% ± 0.2%	0.4% ± 0.3%
$CD56^{dim}CD94^{med}$CD16+CD57- (Percentages)				
NBM	28.1 ± 2.7%	9.8% ± 1.8%	9.7% ± 1.2%	4.9% ± 1.1%
MDS	24.9% ± 3.2%	7.1% ± 1.9%	6.1% ± 0.9%	3.2% ± 0.7%
AML	27.4% ± 4.3%	9.8% ± 3.8%	9.6% ± 3.3%	6.6% ± 3.3%
$CD56^{dim}CD94^{med/low}$CD16+CD57+ (Percentages)				
NBM	11.4% ± 1.3%	10.3% ± 1.8%	12.7% ± 1.6%	5.1% ± 1.4%
MDS	13.9% ± 2.0%	11.1% ± 2.0%	12.9% ± 2.5%	7.2% ± 1.7%
AML	16.7% ± 6.1%	9.7% ± 5.1%	11.1% ± 3.4%	6.2% ± 3.1%

Values represent mean percentages ± SE (standard error) from each group of cases: normal bone marrow (NBM), myelodysplastic syndromes (MDS), acute myeloid leukemia (AML).
